# Australian Ready Meals: Does a Higher Health Star Rating Mean Lower Sodium Content?

**DOI:** 10.3390/nu14061269

**Published:** 2022-03-17

**Authors:** Alyse Davies, Joseph Alvin Santos, Emalie Rosewarne, Anna Rangan, Jacqui Webster

**Affiliations:** 1 Charles Perkins Centre, Nutrition and Dietetics Group, Sydney Nursing School, Faculty of Medicine and Health, University of Sydney, Sydney, NSW 2006, Australia; anna.rangan@sydney.edu.au; 2 The George Institute for Global Health, The University of New South Wales, Sydney, NSW 2006, Australia; jsantos@georgeinstitute.org.au (J.A.S.); erosewarne@georgeinstitute.org.au (E.R.); jwebster@georgeinstitute.org.au (J.W.)

**Keywords:** convenience foods, health star rating, ready meals, prepared meals, sodium content, consumer education

## Abstract

Ready meals are typically a high sodium product and excessive sodium increases the risk for chronic disease. The study aimed to explore the association between sodium content, Health Star Rating (HSR) and the Healthy Food Partnership (HFP) sodium reduction target for ready meals. Median (IQR) sodium content in mg/100 g and mg/serving were determined overall and for each subcategory (ambient, chilled and frozen). Wilcoxon rank sum test was used to compare the sodium content between ready meals with and without HSR. The Jonckheere trend test was used to assess presence of trend in sodium content by HSR categories. In total, 631 ready meals were included and 311 (49%) met the HFP sodium target (<250 mg per 100 g). The percentage of products displaying the voluntary front-of-pack HSR was 52% and of these, 82% had a star rating ≥3.5. A lower median sodium content (mg/100 g) was consistently observed for products with HSR compared with products without HSR (all *p*’s < 0.05). Except for ambient ready meals, a trend was observed where the higher the HSR category, the lower the sodium content (*p* < 0.001). A higher proportion of the products with HSR ≥ 4 met the HFP sodium target for ready meals.

## 1. Introduction

Over the past two decades, there has been a documented shift in Australia’s food practices including reduced time spent on food preparation and cooking and an increased consumption of pre-prepared foods, which include ready meals [1]. Ready meals are defined as “pre-prepared, complete meals that require no extra ingredients and minimal preparation, other than heating” [2]. Perceived time scarcity, value for money, convenience, declining cooking skills, and changes to the family and workplace environments have all played a role in the rise in popularity of these products [1,3,4].

The ready meal market (includes ambient, chilled and frozen products) continues to grow rapidly with the number of products increasing on average by 13% each year [5]. Chilled ready meals are growing at the highest rate [6] and considered to be the most expensive, while ambient products are the least expensive [5]. In Australia, the ready meal market was worth $AUD 853.61 million in 2013, 1.14 billion in 2019 and by 2024, it is expected to be worth $AUD 1.58 billion [7]. Given approximately 75% of dietary sodium is contributed by processed foods (including ready meals) [8], coupled with the growth in the convenience food market, it is important to continually monitor the sodium levels in these products. Sodium is added to ready meals for a variety of reasons, including preservation, taste and texture, and previous studies in Australia and internationally have shown that these products have high sodium levels [2,9,10]. When examining the sodium content in ready meals over time in Australia, there are inconsistencies reported in the literature. One study reported a modest change in sodium density (median 275 mg to 240 mg/100 g) between 2014 to 2020 [5] and others reporting no change between 2008 and 2011 (mean 279 mg versus 277 mg/100 g) [2] and 2010 and 2017 (mean 281 mg versus 282 mg/100 g) [6].

The Food and Health Dialogue, initiated in 2009, set sodium reduction targets for nine food categories, but did not set a target for ready meals. In 2018, the Healthy Food Partnership (HFP) Reformulation Working Group proposed nutrient reformulation targets for 36 food categories, which included a category for ready meals [11]. After public and stakeholder consultations in May 2021, the Reformulation Working Group endorsed a voluntary maximum sodium target for ready meals of 250 mg per 100 g by 30 June 2025 [12,13]. Given excessive dietary sodium is associated with increased blood pressure (BP) and chronic disease risk [14], it is important that the food supply comply with these proposed targets. The World Health Organization’s (WHO) salt intake target has been set at less than 5 g of salt per day (equivalent to 2000 mg of sodium) [15], while the mean daily dietary salt intake for Australians is approximately 9.9 g [16], putting the population at increased risk for chronic disease including hypertension and cardiovascular disease (CVD).

The Health Star Rating (HSR) system was introduced in 2014 and is a voluntary front-of-pack labelling system, rating the nutritional composition of products from half a star to five stars, with five stars being a healthier choice [17]. Ratings are generated using a category-based algorithm where baseline points are determined for energy and nutrients associated with an increased risk of chronic disease including saturated fat, sugar and sodium per 100 g; as well as modifying points for nutrients and/or foods beneficial for health including fibre, protein, fruit, vegetables, nuts and legumes [18,19].

Considering the sodium target for ready meals has recently been endorsed, and no studies have examined the sodium content of meals by the HSR, this study therefore aimed to explore the association between sodium content, Health Star Rating and the Healthy Food Partnership sodium reduction target for ready meals.

## 2. Materials and Methods

### 2.1. Data Collection Process

An annual systematic survey of Australian ready meal products was undertaken in 2019 following a standardised protocol for data collection from six retailers in Sydney, Australia: Woolworths (Town Hall and Marrickville Metro), Coles (Broadway), Aldi (Leichardt), IGA (Pyrmont) and Harris Farm (Broadway) [20]. Combined, Woolworths, Coles, Aldi and IGA account for more than 80% of the Australian grocery market share [21]. Data were captured by taking pictures of the front-of-pack information such as brand name, product name and package size, HSR, the nutrition information panel (NIP), ingredients list and barcode in store. All product information was encoded onto The George Institute’s branded food composition database, FoodSwitch [22]. Ethics approval was not required.

### 2.2. Product Inclusion

**Figure 1:**
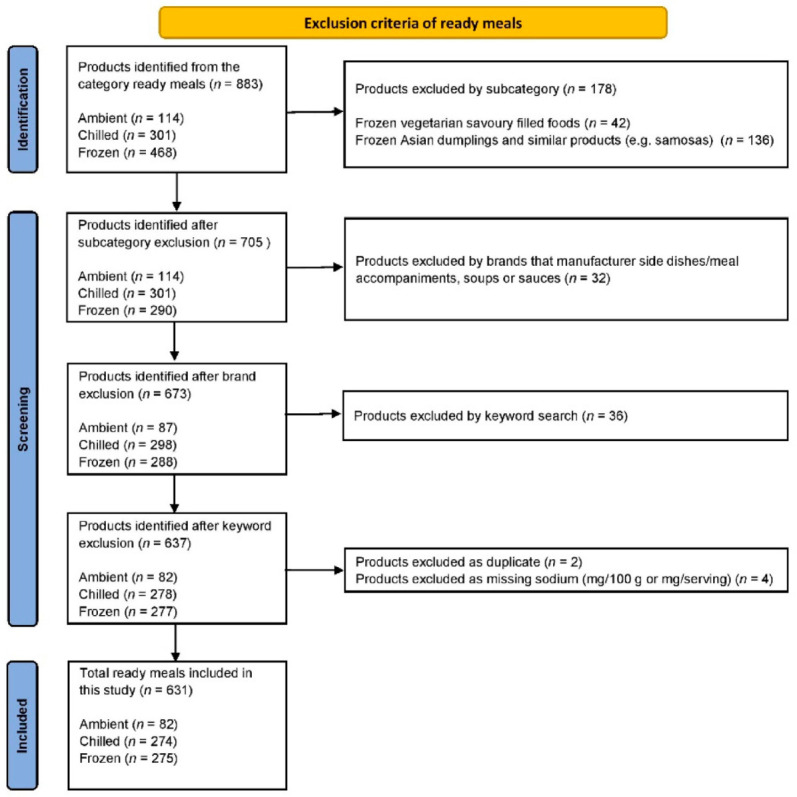
A flow diagram of excluded products.

Ready meals were defined as “pre-prepared, complete meals that required no extra ingredients and minimal preparation, other than heating” [2]. The ready meal category is classified into ambient, chilled and frozen products. The products were screened by one reviewer (A.D.) and exclusions were checked by a second reviewer (E.R.). Initially, exclusions were made by subcategory (e.g., dumplings and similar products), then by brands manufacturing side dishes/meal accompaniments, soups and sauces, then by using a keyword search of the product name (e.g., Arancini, chips, mash, bites). Duplicate products, as well as products with missing sodium (mg/100 g or mg/serving), were screened and excluded. A flow diagram is provided in Figure 1.

### 2.3. Data Analysis

The total product count and product count for each subcategory were recorded. Normality of sodium (mg/100 g and mg/serving size) and serving size (g) was assessed using Shapiro-Wilk Test. Median (IQR) sodium content in mg/100 g and mg/serving were determined overall and for each subcategory: ambient, chilled and frozen ready meals. Wilcoxon rank sum test was used to compare the sodium content and serving size between ready meals with and without a front-of-pack HSR. The Jonckheere trend test was used to assess presence of trend in sodium content by HSR categories (≤3, 3.5, ≥4 stars). Kruskal-Wallis test was conducted to assess the difference in serving size between HSR categories. The difference in the proportion of products meeting the HFP sodium target for ready meals (<250 mg/100 g) was examined using chi-squared tests. Statistical analysis was carried out in IBM SPSS Statistics version 26. A *p*-value of 0.05 was considered as statistically significant.

## 3. Results

### 3.1. All Ready Meals and Subcategories

In total, 631 ready meals were included in this study, further categorised into ambient (*n* = 82, 13%), chilled (*n* = 274, 43%) and frozen (*n* = 275, 44%) products (Table 1). For all ready meals, the median (IQR) sodium content (mg/100 g) was 250 mg (123) and sodium content (mg/serving) was 763 mg (325). In total, 311 (49%) ready meals met the HFP sodium target. Chilled products had the lowest median sodium (mg/100 g and mg/serving) and a higher proportion of products met the sodium target.

### 3.2. Comparison of Products with and without HSR

The comparison of sodium content (mg/100 g and mg/serving) for products with and without HSR and the proportion of products meeting the HFP sodium target are shown in Table 1. Overall, 329 (52%) products displayed HSR, which was further classified into: ambient (*n* = 43, 52%), chilled (*n* = 122, 45%) and frozen (*n*= 164, 60%) ready meals. A lower median sodium content (mg/100 g) was consistently observed for products with HSR compared with products without HSR (all *p*’s < 0.05). In terms of sodium per serving, chilled ready meals with HSR had lower median sodium compared with products without HSR (*p* = 0.011). Apart from frozen ready meals, all categories that displayed HSR had a higher proportion of products meeting the HFP sodium target.

### 3.3. Comparison by Health Star Rating

Table 2 shows the comparison of sodium content (mg/100 g and mg/serving size) by HSR categories (≤3, 3.5 and ≥4 stars) and the proportion of products meeting the HFP sodium target. The majority of ready meals (82%) had a star rating ≥3.5. For all ready meals combined, as well as the chilled and frozen sub-categories, there was a trend in sodium content (mg/100 g and mg/serving), where the higher the HSR category, the lower the sodium content and the higher the proportion of products meeting the sodium target.

## 4. Discussion

The present study explored the association between sodium content, HSR and the new HFP sodium target for ready meals. The percentage of products displaying the voluntary front-of-pack HSR was 52% and of these, 82% had a star rating ≥3.5. The products that displayed a HSR had a lower median sodium and a greater proportion of these products met the sodium target. Except for ambient ready meals, a trend in sodium content was observed, where the higher the HSR, the lower sodium content (mg/100 g and mg/serving). Products with a HSR ≥ 4 were more likely to meet the HFP sodium target.

Although research has shown that Australian consumers are willing to pay more for products with a HSR [23], given the voluntary nature of the HSR system, not all products display the HSR, making it harder for consumers to make informed food choices. Just over half of the ready meal products identified in our annual supermarket survey displayed HSR (52%), and a previous study reported a trend towards less products displaying the HSR since 2016 [5]. In 2019, the HSR system five-year report was published and manufactures provided reasons for not adopting the HSR [19]. The three overarching themes included (1) the undermined confidence in the HSR system; (2) the lack of commercial incentives and consumer demand for the HSR system and (3) the uncertainty around the likelihood of the HSR continuing. While there has been consideration of mandatory HSR, state and federal food ministers decided against this, and will continue to be voluntary with focus on increasing participation by food industry (70% across intended products by 2025) [19,24]. Increased efforts and incentives for manufacturers to display HSR is required to ensure uptake targets are reached by 2025.

This study supports previous research showing products with healthier nutritional profiles are more likely to show a HSR than unhealthy products [25,26]. In our study, we found that products with HSR had significantly lower median sodium per 100 g than those without HSR. These findings are similar to a study using data from 2016 (two years after the adoption of HSR), that examined all packaged foods and found products displaying HSR had significantly lower mean sodium per 100 g [27]. Additionally, our study assessed the sodium content of ready meals across HSR categories to understand if a higher HSR was associated with lower sodium given the HSR algorithm considers energy, other negative components (saturated fat and sugar) and positive components (protein, dietary fibre and the proportion of fruits, vegetables, nuts and legumes). A trend was observed for all ready meals as well as chilled and frozen sub-categories, where the higher the HSR, the lower the sodium content. No trend was observed for ambient ready meals, which may due to the small amount of products in each HSR category. Ambient products had high sodium content in general, which suggests that sodium may be used as a preservative in this category. Based on these results, we can confirm that even though the HSR algorithm is multifaceted and complex in nature, the HSR system performs well in terms of sodium content across categories.

While the HFP sodium targets were developed taking into account the sodium cut off levels for the HSR algorithm, the results of this work suggest there is a small degree of misalignment. Although a large proportion of the products ≥4 stars met the sodium target (83%), the majority of products (57%) had a >3.5 star rating and only 53% of these met the sodium target for ready meals. Whilst previous reports have suggested that products ≥3.5 stars are a healthier choice [28,29], it is clear that products in this star category may not be “healthy” with regards to sodium content. Our findings suggests that consumers should aim to purchase products with at least 4 stars, especially individuals with high BP or CVD, as these products contained less sodium, and the majority met sodium targets. In terms of sub-categories, chilled ready meals with a star rating of ≥4 had the lowest sodium content, with a median of 177 mg/100 g and 90% of products met the sodium target. A total of 80% of frozen ready meals with a star rating of ≥4 met the HFP sodium target however, for ambient ready meals, even products ≥4 stars were unlikely to meet the HFP sodium target.

Nutritional labelling schemes, such as the HSR scheme, have been identified as an incentive for industry to reformulate their products to contain healthier nutritional profiles [27,30]. Previous research has reported a 1.4% sodium change [25] and a 49 mg/100 g sodium decrease [27] once manufacturers have adopted the HSR, which indicates that the HSR is driving reformulation. Our results show that there is a wide range in the sodium content per serving, which indicates that there is still additional scope for manufactures to reformulate products to contain less sodium. Further, a chilled ready meal without a front-of-pack HSR contained 6990 mg of sodium per serving, which is more than three times over the WHO daily-recommended maximum in one meal and it could be argued that this product should contain a health warning. Reducing the amount of sodium in commonly consumed convenience foods through food reformulation can help to improve the population’s diets as the burden of diet-related chronic diseases are high [31].

The present study was the first to investigate the sodium content of ready meals in terms of the HSR. A representative sample of ready meals were obtained from supermarkets in Australia with over 80% of the market share. This study was not without limitations. Only half of the products identified displayed HSR, which resulted in a small number of products in each HSR category. No laboratory analyses were undertaken and all data were derived from the NIP. The data were collected in 2019 and due to the COVID-19 pandemic, data collection was not possible for 2020.

## 5. Conclusions

Products with a HSR had lower sodium content which supports the hypothesis that the voluntary use of the HSR means manufacturers are more likely to display HSR on healthier products. Even though the HSR algorithm is multifaceted and complex in nature, the HSR system performs well in terms of sodium content across categories. The majority of products had a 3.5 star rating, but only half of these met the HFP sodium target, and therefore this category may not be “healthy” in terms of sodium content. The subcategory that performed the best in terms of the lowest sodium content and the highest proportion of products meeting the sodium target was the chilled category. A higher proportion of the products with HSR ≥ 4 met the HFP sodium target for ready meals.

## Figures and Tables

**Table 1:** Comparison of median (IQR) sodium content of products with and without a front-of-pack Health Star Rating, and proportion meeting the Healthy Food Partnership sodium reduction target (<250 mg/100 g).

Ready Meals 2019		Health Star Rating		
	All Ready Meals	Yes	No	*p*-Value
All ready meals, *n* (%)	631 (100)	329 (52)	302 (48)	
Serving size (g), median (IQR)	340 (90)	350 (95)	320 (100)	<0.001
Sodium content (mg/100 g), median (IQR)	250 (123)	238 (91)	274 (152)	<0.001
Sodium content (mg/serving), median (IQR)	763 (325)	753 (286)	772 (425)	0.134
Proportion < 250 mg/100 g, *n* (%)	311 (49)	184 (56)	127 (42)	<0.001
Ambient ready meals, *n* (%)	82 (13)	43 (52)	39 (48)	
Serving size (g), median (IQR)	213 (151)	250 (160)	206 (130)	0.780
Sodium content, mg/100 g, median (IQR)	300 (174)	264 (113)	356 (155)	0.001
Sodium content, mg/serving, median (IQR)	735 (372)	725 (481)	757 (446)	0.059
Proportion < 250 mg/100 g, *n* (%)	21 (26)	16 (37)	5 (13)	0.012
Chilled ready meals, *n* (%)	274 (43)	122 (45)	152 (55)	
Serving size (g), median (IQR)	350 (75)	350 (106)	330 (73)	0.020
Sodium content, mg/100 g, median (IQR)	233 (145)	217 (106)	249 (160)	0.010
Sodium content, mg/serving, median (IQR)	730 (400)	683 (294)	795 (576)	0.011
Proportion < 250 mg/100 g, *n* (%)	157 (57)	81 (66)	76 (50)	0.006
Frozen ready meals, *n* (%)	275 (44)	164 (60)	111 (40)	
Serving size (g), median (IQR)	350 (75)	350 (80)	320 (100)	<0.001
Sodium content, mg/100 g, median (IQR)	251 (88)	240 (77)	274 (117)	0.017
Sodium content, mg/serving, median (IQR)	791 (315)	799 (305)	767 (345)	0.251
Proportion < 250 mg/100 g, *n* (%)	133 (48)	87 (53)	46 (41)	0.059

**Table 2:** Comparison of median (IQR) sodium content of products using the Health Star Rating on the front-of-pack and proportion meeting the Healthy Food Partnership sodium reduction target (<250 mg/100 g).

Ready Meals 2019		Health Star Rating (HSR)			
	No HSR	≤3 Stars	3.5 Stars	≥4 Stars	*p*-Value
All ready meals, *n* (%)	302 (48)	60 (18)	188 (57)	81 (25)	
Serving size (g), median (IQR)	320 (100)	335 (186)	350 (94)	350 (80)	0.212
Sodium content, mg/100 g, median (IQR)	274 (152)	280 (154)	240 (83)	190 (86)	<0.001
Sodium content, mg/serving, median (IQR)	772 (425)	943 (524)	770 (237)	634 (249)	<0.001
Proportion <250 mg/100 g, *n* (%)	127 (42)	17 (28)	100 (53)	67 (83)	<0.001
Ambient ready meals, *n* (%)	39 (48)	12 (28)	25 (58)	6 (14)	
Serving size (g), median (IQR)	206 (130)	206 (209)	250 (144)	195 (175)	0.452
Sodium content, mg/100 g, median (IQR)	356 (155)	240 (199)	275 (114)	274 (152)	0.365
Sodium content, mg/serving, median (IQR)	757 (446)	734 (670)	729 (310)	574 (395)	0.284
Proportion <250 mg/100 g ^†^, *n* (%)	5 (13)	7 (58)	7 (28)	2 (33)	0.221
Chilled ready meals, *n* (%)	152 (55)	18 (15)	54 (44)	50 (41)	
Serving size (g), median (IQR)	330 (73)	275 (108)	350 (79)	350 (50)	0.002
Sodium content, mg/100 g, median (IQR)	249 (160)	283 (230)	232 (100)	177 (80)	<0.001
Sodium content, mg/serving, median (IQR)	795 (576)	820 (434)	729 (260)	626 (278)	<0.001
Proportion <250 mg/100 g, *n* (%)	76 (50)	4 (22)	32 (59)	45 (90)	<0.001
Frozen ready meals, *n* (%)	111 (40)	30 (18)	109 (66)	25 (15)	
Serving size (g), median (IQR)	320 (100)	388 (85)	350 (75)	350 (73)	0.066
Sodium content, mg/100 g, median (IQR)	274 (117)	300 (128)	238 (77)	223 (67)	<0.001
Sodium content, mg/serving, median (IQR)	767 (345)	1058 (538)	795 (205)	689 (216)	<0.001
Proportion <250 mg/100 g, *n* (%)	46 (41)	6 (20)	61 (56)	20 (80)	<0.001

^†^ Fishers Exact Test.

## Data Availability

The datasets used and/or analysed during the current study available from the corresponding author on reasonable request.
